# The effect of daily oral PrEP use during pregnancy on bone mineral density among adolescent girls and young women in Uganda

**DOI:** 10.3389/frph.2023.1240990

**Published:** 2024-01-08

**Authors:** Kidist Zewdie, Flavia M. Kiweewa, Timothy Ssebuliba, Susan A. Morrison, Timothy R. Muwonge, Jade Boyer, Felix Bambia, Josephine Badaru, Gabrielle Stein, Kenneth K. Mugwanya, Christina Wyatt, Michael T. Yin, Andrew Mujugira, Renee Heffron

**Affiliations:** ^1^ Department of Epidemiology, University of Washington, Seattle, WA, United States; ^2^ Department of Global Health, University of Washington, Seattle, WA, United States; ^3^ Makerere University-Johns Hopkins University Research Collaboration, Kampala, Uganda; ^4^ Infectious Diseases Institute, Makerere University, Kampala, Uganda; ^5^ Department of Medicine, Division of Infectious Disease, Columbia University, New York, NY, United States; ^6^ Department of Medicine, Division of Nephrology, Duke University School of Medicine, Durham, NC, United States; ^7^ Department of Medicine, University of Alabama at Birmingham, Birmingham, AL, United States

**Keywords:** bone mineral density, oral PrEP, young women and adolescent girls, HIV prevention, Uganda

## Abstract

**Introduction:**

Oral pre-exposure prophylaxis (PrEP) is recommended during pregnancy for at-risk cisgender women. Pregnancy is known to impede bone growth and tenofovir-based PrEP may also yield detrimental changes to bone health. Thus, we evaluated the effect of PrEP use during pregnancy on bone mineral density (BMD).

**Methods:**

We used data from a cohort of women who were sexually active, HIV-negative, ages 16–25 years, initiating DMPA or choosing condoms for contraception and enrolled in the Kampala Women's Bone Study. Women were followed quarterly with rapid testing for HIV and pregnancy, PrEP dispensation, and adherence counseling. Those who became pregnant were counseled on PrEP use during pregnancy per national guidelines. BMD of the neck of the hip, total hip, and lumbar spine was measured using dual-energy x-ray absorptiometry at baseline and annually. We compared the mean percent change in BMD from baseline to month 24.

**Results:**

Among 499 women enrolled in the study, 105 pregnancies occurred in 90 women. At enrollment, the median age was 20 years (IQR: 19–21) and 89% initiated PrEP. During pregnancy, 67% of women continued using PrEP and PrEP was dispensed in 64% of visits. BMD declined significantly in women using PrEP during pregnancy compared to women who were not pregnant nor used PrEP: relative BMD change was −2.26% (95% CI: −4.63 to 0.11, *p* = 0.06) in the femoral neck, −2.57% (95% CI: −4.48 to −0.66, *p* = 0.01) in total hip, −3.06% (95% CI: −5.49 to −0.63, *p* = 0.001) lumbar spine. There was no significant difference in BMD loss when comparing PrEP-exposed pregnant women to pregnant women who never used PrEP. Women who became pregnant were less likely to continue PrEP at subsequent study visits than women who did not become pregnant (adjOR: 0.25, 95% CI: 0.16–0.37, *p* < 0.001). Based on pill counts, there was a 62% reduction in the odds of high PrEP adherence during pregnancy (adjOR = 0.38, 95% CI: 0.27–0.58, *p* < 0.001).

**Conclusion:**

Women who used PrEP during pregnancy experienced a similar reduction in BMD as pregnant women with no PrEP exposure, indicating that BMD loss in PrEP-using pregnant women is largely driven by pregnancy and not PrEP.

## Introduction

Pregnancy is a period with an elevated risk for acquiring HIV (1–3), estimated to be >2-fold higher than non-pregnant periods (1, 4). Biological changes in hormonal levels as well as changes in sexual behavior are likely responsible for the increase in HIV susceptibility of cisgender women during pregnancy (5, 6). Pregnancy rates in sub-Saharan Africa are among the highest in the world and oral PrEP can play a critical role in reducing HIV acquisition during this period (7, 8). Oral PrEP containing tenofovir disoproxil fumarate (TDF) is safe and recommended for use during pregnancy and postpartum by women at substantial risk of acquiring HIV (9–11).

With reassuring data on the safety of PrEP with regards to birth outcomes and infant growth (10), the remaining questions are related to whether there are subclinical consequences from PrEP use during pregnancy, such as effects on bone health. Women's bone mineral density (BMD) reaches its peak between the ages of 20 and 26 years and plateaus until menopause (12, 13). However, BMD loss or premature attainment of peak BMD can occur in premenopausal women due to various reasons, including the use of depot medroxyprogesterone acetate (DMPA), pregnancy, and breastfeeding (12). Changes in BMD during pregnancy and lactation are due to mineral transfer to a fetus or infant to facilitate growth (12, 14). Additionally, the use of TDF-based oral PrEP has been postulated to be a potential factor linked to BMD loss (15, 16) because of its excretion through the renal system and the kidney-bone development pathway (17, 18). Despite the independent association of pregnancy and BMD and the subclinical impact of TDF on creatinine levels, it is not known whether PrEP use during pregnancy and/or breastfeeding could exacerbate BMD loss in young women.

In addition, how pregnancy impacts oral PrEP adherence and continuation needs to be further evaluated. A recent PrEP implementation study among pregnant women found that only 40% continued PrEP use one month after initiation (19). While protecting the fetus from HIV might provide an incentive for pregnant women to use and adhere to PrEP, experiencing side effects in conjunction with those elicited by pregnancy and fear of unknown effects on the fetus might prompt discontinuation, beyond the effects of stigma and pill burden that all PrEP users face (19–23). Prior studies have primarily examined patterns of PrEP use among women who initiated PrEP use during pregnancy; however, PrEP use patterns may differ in women who were already on PrEP at the time of pregnancy.

Using data from women enrolled in a prospective cohort study evaluating the impact of concurrent TDF-based PrEP and DMPA on bone health in Kampala, Uganda, we evaluated the impact of TDF-based PrEP use on BMD loss during pregnancy. Secondarily, we investigated the effect of pregnancy on daily oral PrEP adherence and continuation.

## Methods

### Study design and population

We used data from all women enrolled in the Kampala Women's Bone Study (ClinicalTrials.gov #NCT03464266), an open-label prospective cohort study aimed to address bone safety questions with concurrent TDF-based PrEP and DMPA use. Between May 2018 and March 2020, the Kampala Women's Bone Study recruited women who were at high risk for HIV and seeking DMPA or condoms as contraception in family planning clinics, youth-based centers, and higher learning institutions in Kampala, Uganda. Women who were HIV-negative, ages 16–25 years, initiating DMPA or choosing to use male condoms for contraception, without contraindications for DMPA or TDF-based PrEP, and not planning to become pregnant in the next 24 months were eligible to enroll in the study.

### Data collection and outcomes

Over 24 months, women were followed quarterly with HIV prevention counseling and condom distribution, diagnostic testing for HIV (using rapid testing according to the national algorithm), urine pregnancy testing, provision of DMPA injections, offers of PrEP, PrEP adherence counseling, and provision of PrEP medication (FTC/ TDF). At enrollment and quarterly visits, interviewers administered standardized questionnaires to collect data on demographic characteristics, medical history, sexual behavior, sexual relationship power, HIV perception and salience, diet and physical activity, alcohol and drug use, and contraceptive and PrEP use. At the first visit at which the participant was found to be pregnant, data on the last menstrual period date, expected delivery date, whether the pregnancy was intended, obstetric history, and decision on PrEP continuation were collected. Women who became pregnant while using PrEP were counseled about the known and unknown risks and benefits of PrEP use during pregnancy according to the national guidelines and supported to continue or discontinue PrEP.

At enrollment and annual study visits, after confirming HCG negative urine pregnancy test results, dual-energy x-ray absorptiometry (DXA) scans were conducted to measure BMD for the lumbar spine, total hip, and neck of the hip. For women who were pregnant, DXA scans were withheld and completed as soon after pregnancy as possible. We measured PrEP continuation using pharmacy PrEP refill data and pill count as measures of PrEP adherence and defined “continuation” based on PrEP being dispensed at the visit. Quarterly pill use was quantified by dividing the number of pills used and pills not returned by the expected number of pills to be used, and a value of ≥80% was considered high adherence. The start of pregnancy was estimated using the last menstrual period date or the estimated delivery dates. The end of pregnancy was determined using the reported date of pregnancy outcome or estimated delivery date.

### Statistical analysis

Baseline participant characteristics were summarized using descriptive statistics. To evaluate the effect of PrEP use during pregnancy on BMD, we used a generalized linear model (GLM) with a Gaussian link to compare the mean percent change in BMD between baseline and the end of the two-year follow-up in women who were using PrEP during pregnancy and non-pregnant women who didn't initiate PrEP during the study. Models were adjusted for confounders identified *a priori*: age as a continuous variable, baseline body-mass index (BMI), and baseline DMPA use. In a sensitivity analysis, we repeated the analysis excluding non-full-term pregnancies. To evaluate the effect of pregnancy on PrEP continuation and PrEP adherence, we used generalized estimation equation (GEE) models with a logit-link and exchangeable correlation structure to compare the odds of PrEP continuation and PrEP adherence between women who experience pregnancy and those who did not experience pregnancy over the 24 months study follow up. The models were adjusted for potential confounders identified *a priori*: age, education, income, relationship status, and partner's HIV status. In separate models, we compared PrEP continuation during pregnancy to non-pregnant periods among women who became pregnant during the study. All analyses were done using R 4.0.

### Ethical considerations

The study protocol was approved by the National HIV/AIDS Research Committee of Uganda, the Uganda National Council for Science and Technology, and the Human Subjects Division at the University of Washington. Participants ≥18 years provided written informed consent and participants <18 years provided written assent with a consenting guardian or were qualified to provide consent based on their status as an emancipated or mature minor.

## Results

### Participant characteristics

A total of 499 sexually active young women were enrolled in the study. At enrollment, the median age was 20 years [interquartile range (IQR):19–21], 87 were married or had a steady partner, 92% received financial support from their partners, 63% did not know their partner's HIV status, and 89% initiated PrEP. Over the 24-month study period, 90 participants became pregnant. Women who became pregnant more frequently had chosen to use condoms than DMPA at baseline as a contraceptive compared to women who did not become pregnant (61% vs. 43%, respectively). Other baseline characteristics including age, marital status, education level, sexual behavior characteristics, BMI, and BMD were similar between women who did and did not become pregnant (Table 1).

**Table 1:** Baseline characteristics of women in the study (*N* = 499).

Characteristic	No pregnancy during study, *N* = 409, *N* (%) or median (IQR)	At least one pregnant during study follow-up, *N* = 90, *N* (%) or median (IQR)	Total, *N* = 499, *N* (%) or median (IQR)
Age (years)	20 (19, 21)	20 (18, 21)	20 (19, 21)
Relationship status			
Single	48 (12%)	17 (19%)	65 (13%)
Married/in a steady partnership	361 (88%)	73 (81%)	434 (87%)
Lives with partner	22 (5.4%)	2 (2.2%)	24 (4.8%)
Earns own income	211 (52%)	50 (56%)	261 (52%)
Partner provides financial support	377 (92%)	80 (89%)	457 (92%)
Years of education	11 (8, 12)	11 (9, 12)	11 (8, 12)
Partners HIV status			
Positive	7 (1.7%)	2 (2.2%)	9 (1.8%)
Negative	147 (36%)	30 (33%)	177 (36%)
Unknown	254 (62%)	58 (64%)	312 (63%)
Travel time to research clinic			
<1 h	58 (14%)	18 (20%)	76 (15%)
1–2 h	335 (82%)	68 (76%)	403 (81%)
>2 h	16 (3.9%)	4 (4.4%)	20 (4.0%)
Any condomless sex, past 3 months	274 (67%)	63 (70%)	337 (68%)
Any condomless sex, past 7 days	123 (49%)	23 (47%)	146 (48%)
Had more than one partner, past 3 months	238 (58%)	41 (46%)	279 (56%)
Contraception choice			
Condoms	176 (43%)	55 (61%)	231 (46%)
DMPA	233 (57%)	35 (39%)	268 (54%)
Initiated PrEP	360 (88%)	80 (89%)	431 (86%)
Body-mass index, kg/m²	23 (21, 25)	23 (21, 25)	22 (21, 25)
Mean BMD (g/cm^2^)			
The neck of the hip	0.86 (0.11)	0.87 (0.12)	0.85 (0.11)
Lumbar spine	0.95 (0.12)	0.95 (0.12)	0.93 (0.11)
Total hip	0.94 (0.10)	0.94 (0.10)	0.93 (0.09)

### Pregnancy characteristics

Among 499 participants enrolled in the study, 396 (79%) were retained for one year, and 331 (66%) participants were followed for two years. Although we were not able to contact the majority (60%) of participants who were lost to follow-up to ascertain reasons for study discontinuation, two-thirds of the loss to follow-up occurred after March 2020, when the COVID-19 pandemic began in Uganda. During the study period, 105 pregnancies occurred, including 15 women who experienced multiple pregnancies. The median time between enrolment and the start of pregnancy was 426 days (IQR: 235–524). Among those who became pregnant, 61 (67%) women [during 72 (69%) pregnancies] used PrEP during their pregnancy (Table 2). Overall, 73% of pregnancies were unintended, 62% were the woman's first pregnancy, and 35% of pregnancies resulted in pregnancy loss. There was no difference in pregnancy outcomes by PrEP exposure groups.

**Table 2:** Pregnancy characteristics.

		Used PrEP during pregnancy	
Characteristic	Overall, *N* = 105, *n* (%)	No, *N* = 33, *n* (%)	Yes, *N* = 72, *n* (%)
Pregnancy was intended^a^			
No	77 (73%)	19 (58%)	58 (81%)
Yes	28 (27%)	14 (42%)	14 (19%)
Number of previous pregnancies			
None	65 (62%)	20 (61%)	45 (62%)
One	33 (31%)	11 (33%)	22 (31%)
More than one	7 (7%)	2 (6%)	5 (7%)
Pregnancy outcome			
Live birth	40 (38%)	12 (38%)	28 (42%)
Premature live birth	3 (3.0%)	0 (0%)	3 (4%)
Pregnancy loss	37 (35%)	12 (38%)	25 (37%)
Unknown	25 (24%)	9 (27%)	16 (22%)

^a^ Ascertained through interviewer conversation with the participant.

### Association of PrEP use, pregnancy, and bone mineral density

We examined the association between PrEP use during pregnancy with changes in mean BMD from baseline to 2 years at the neck of the hip, lumbar spine, and total spine. Among the 331 study participants who were followed for two years, 294 (89%) participants had DXA scans at baseline and the 24-month visit. The median time between the end of pregnancy and the exit DXA scan was 119 days [IQR: 55–221]. The mean percent change in BMD for pregnant women who used PrEP during pregnancy at the neck of the hip was −1.91% (95% CI: −4.28% to +0.46%), −2.20% (95% CI: −4.17% to −0.23%) at the total hip and −3.78% (95% CI: −6.28% to −1.27%) at the lumbar spine [Table 3]. Over the 24-month study period, the mean percent change in BMD was significantly greater in pregnant women using PrEP during pregnancy relative to women who were not exposed to either PrEP or pregnancy. After adjusting for age, BMI, and DMPA use prior to pregnancy, the relative mean percent change in BMD was −2.26% (95% CI: −4.63 to 0.11, *p* = 0.06) at the femoral neck, −2.57% (95% CI: −4.48 to −0.66, *p* = 0.01) at the total hip, and −3.06% (95% CI: −5.49 to −0.63, *p* = 0.001) at the lumbar spine. The decline in BMD in those pregnant but who had never been exposed to PrEP or who were pregnant but not taking PrEP during pregnancy was not significantly different compared to women who were not pregnant and had never been on PrEP, although numbers were small in both groups.

**Table 3:** Adjusted difference in the mean BMD at the neck of the hip, lumbar spine, and total hip .

			Comparisons to women who never pregnant and never used PrEP		Comparisons of the impact of pregnancy among women who used PrEP		Comparisons of the impact of PrEP among women who experienced pregnancy	
	*N* = 294	% Change in BMD from baseline (g/cm^2^)	Adjusted difference in % change in BMD (95% CI)^a^	*p*-value	Adjusted difference in % change in BMD (95% CI)^a^	*p*-value	Adjusted difference in % change in BMD (95% CI)^a^	*p*-value
The neck of the hip (g/cm^2^)								
Not pregnant and no PrEP use ever	31	0.12 (−1.64, 1.89)	Ref.		—		—	
Not pregnant and used PrEP	206	0.54 (−1.35, 2.44)	0.21 (−1.75, 2.16)	0.83	Ref.		—	
Pregnant, no PrEP ever	6	−0.08 (−4.47, 4.31)	0.01 (−4.33, 4.33)	0.99	—		Ref	
Pregnant, no PrEP during pregnancy	12	−1.82 (−5.17, 1.53)	−1.59 (−5.03, 1.84)	0.36	—		—	
Pregnant and PrEP use during pregnancy	39	−1.91 (−4.28, 0.46)	−2.26 (−4.63, 0.11)	0.06	−2.47 (−4.22, −0.71)	0.006	−2.26 (−6.54, 2.01)	0.30
Total hip (g/cm^2^)								
Not pregnant and no PrEP use ever	31	1.01 (−0.45, 2.48)	Ref.		—		—	
Not pregnant and used PrEP	206	−0.14 (−1.72, 1.43)	−0.49 (−2.07, 1.09)	0.54	Ref.		—	
Pregnant, no PrEP ever	6	−0.08 (−3.73, 3.57)	−0.10 (−3.60, 3.39)	0.95	—		Ref.	
Pregnant, no PrEP during pregnancy	12	−0.69 (−3.44, 2.13)	−0.78 (−3.55, 2.00)	0.58	—		—	
Pregnant and PrEP during pregnancy	39	−2.20 (−4.17, −0.23)	−2.57 (−4.48, −0.66)	0.01	−2.08 (−3.50, −0.66)	0.004	−2.47 (−5.92, 0.99)	0.16
Lumbar spine (g/cm^2^)								
Not pregnant and no PrEP use ever	31	3.09 (1.22, 4.96)	Ref.		—		—	
Not pregnant and used PrEP	206	−0.50 (−2.50, 1.49)	−0.08 (−2.09, 1.92)	0.95	Ref.		—-	
Pregnant, no PrEP ever	6	−4.05 (−8.68, 0.58)	−3.73 (−8.17, 0.76)	0.11	—		Ref.	
Pregnant, no PrEP during pregnancy	12	−1.75 (−5.28, 1.78)	−0.32 (−3.85, 3.20)	0.60	—		—	
Pregnant and PrEP use during pregnancy	39	−3.78 (−6.28, −1.27)	−3.06 (−5.49, −0.63)	0.01	−2.98 (−4.78, −1.18)	0.001	0.67 (−3.71, 5.06)	0.76

^a^ Adjusted for age, BMI and DMPA use at enrollment.

BMD declined significantly in pregnant women who used PrEP during pregnancy compared to women who used PrEP but did not become pregnant. After adjusting for age, BMI, and DMPA use, the relative mean BMD percent change was −2.47% (95% CI: −4.22 to −0.71, *p* = 0.006) at the femoral neck, −2.08% (95% CI: −3.50 to −0.66, *p* = 0.004) at the total hip, and −2.98% (95% CI: −4.78 to −1.18, *p* = 0.001) at the lumbar spine. The decline in BMD in pregnant women who were using PrEP during pregnancy was not statistically significant compared to women who experienced pregnancy but were not exposed to PrEP. The relative mean BMD percent change was −2.26% (95% CI: −6.54 to 2.01, *p* = 0.30) at the femoral neck, −2.47% (95% CI: −5.92 to 0.99, *p* = 0.16) at the total hip, and 0.67% (95% CI: −3.71 to −5.06, *p* = 0.76) at the lumbar spine. Similar results were observed in a sensitivity analysis limited to full-term pregnancies.

### Prep continuation during pregnancy

Among the 90 women who became pregnant during the study, 10 (11%) did not use PrEP during the study, 19 (21%) did not continue PrEP use during pregnancy, and 61 (67%) chose to continue PrEP during their pregnancy. Among 80 women who became pregnant after initiating PrEP, PrEP was dispensed in 64% of visits during pregnancy (Table 4).

**Table 4:** The association between PrEP continuation and pregnancy.

	PrEP was not dispensed	PrEP dispensed	Unadjusted analysis		Adjusted analysis^a^	
PrEP continuation among pregnant and non-pregnant women in the study						
	Total Visits *N* = 443, *N* (%)	Total Visits, *N* = 3,038, *N* (%)	OR (95% CI)	*p*-value	OR (95% CI)	*p*-value
Pregnant	49 (36%)	87 (64%)	0.32 (0.22–0.46)	<0.001	0.25 (0.17–0.37)	<0.001
Not pregnant	394 (11%)	2,951 (88%)	Ref.	Ref.	Ref.	Ref.
PrEP continuation among women who became pregnant during pregnant and non-pregnant periods						
	Total Visits, *N* = 147, *N* (%)	Total Visits, *N* = 565, *N* (%)				
Pregnant	49 (36%)	87 (64%)	0.38 (0.26–0.56)	<0.001	0.30 (0.20–0.46)	<0.001
Not pregnant	98 (18%)	447 (82%)	Ref.	Ref.	Ref.	Ref.

^a^ Adjusted for age, income, education, partner's HIV status, and relationship status.

After adjusting for age, education, relationship status, income, and partner's HIV status, we found that women who became pregnant were less likely to get PrEP refill at subsequent study visits than women who did not become pregnant (adjusted OR: 0.25, 95% CI: 0.17, 0.37, *p* < 0.001). In the subset of women who became pregnant and had initiated PrEP (*N* = 80), there was a statistically significant 70% reduction in the odds of PrEP continuation during pregnancy (adjusted OR = 0.30, 95% CI 0.20–0.46 *p* < 0.001) compared to their non-pregnant periods.

### Prep adherence

Over the 24-month follow-up period, there were 2,735 follow-up study visits among participants who were dispensed PrEP at a previous visit. Based on pill counts, high PrEP adherence (>80% of expected pills not returned) was reported in 69% of follow-up visits (Table 5). After adjusting for age, education, relationship status, income, and partner's HIV status, women had 62% reduced odds of high PrEP adherence (adjOR 0.38; 95% CI 0.27–0.58, *p* < 0.001) during pregnancy compared to non-pregnant periods.

**Table 5:** Association of PrEP adherence with pregnancy.

	High PrEP adherence	Low PrEP Adherence	Unadjusted analysis		Adjusted analysis^a^	
	Total Visits *N* = 1,878 (*N* %)	Total Visits, *N* = 857 (*N* %)	OR (95% CI)	*p*-value	OR (95% CI)	*p*-value
Pregnant	46 (44%)	58 (56%)	0.36 (0.25–0.54)	<0.001	0.38 (0.27–0.58)	<0.001
Not pregnant	1,832 (70%)	799 (30%)	Ref.	Ref.	Ref.	Ref.

^a^ Adjusted for age, income, education, partner's HIV status, and relationship status.

## Discussion

In this study in Uganda with young women who initiated PrEP before pregnancy, we observed significant BMD loss among pregnant women using PrEP that was likely driven by pregnancy, rather than PrEP use. Our study also reported that women experiencing pregnancy were significantly less likely to use PrEP than women without a pregnancy through analyses of pregnant vs. non-pregnant women and pregnant and non-pregnant periods among women who become pregnant. Additionally, we found that women are less likely to be adherent to PrEP during pregnancy based on pill count data.

Over the two-year follow-up period, we observed a significantly greater loss in BMD among PrEP-exposed pregnant women compared to women who did not become pregnant and were not exposed to PrEP. Isolating our analysis to estimate the effect of PrEP only, we did not see a significant difference in BMD loss when comparing PrEP-exposed pregnant women to pregnant women who never used PrEP. However, it is important to note that in both the femur and the hip, we saw a trend toward a greater reduction in BMD in women who use PrEP during pregnancy, and due to the small sample size of pregnant women who are not exposed to PrEP our estimates may be unstable. Given that previous studies have shown that TDF-based PrEP is associated with bone loss (16, 24, 25) and our study included young women who have not yet achieved peak bone mass, have high fertility rates, and are more likely to be exposed to injectable contraceptives that may compound bone loss (26), any significant BMD reduction in this group is particularly concerning and warrants further investigation. Studies are needed to determine the clinical implications of the decline in BMD associated with concurrent pregnancy and high adherence to TDF-based PrEP in young women and whether the decline is reversible after the end of pregnancy. It is also important to study the potential implications of a more prolonged decline in BMD when TDF-based oral PrEP is used during breastfeeding and the trajectory of BMD subsequent to the cessation of lactation.

Among the 80 women who initiated PrEP and became pregnant, 61(76%) chose to continue PrEP during pregnancy. However, our results indicate at subsequent visits, pregnant women were less likely to get PrEP refills compared to non-pregnant women, highlighting the importance of open discussion about the risks and benefits of PrEP use during pregnancy, the increased risk of HIV acquisition and devising strategies to support prevention-effective PrEP use in adolescent girls and young women during pregnancy. A recent study in South Africa found that the most common reason for PrEP discontinuation among pregnant women was gastrointestinal side effects, including nausea and vomiting (27). Providing women with counseling and strategies to manage nausea and vomiting could improve PrEP continuation. In addition, strategies such as regular adherence counseling, drug-level feedback, and adherence support clubs could be used to support oral PrEP adherence in young pregnant women (28–30).

Research in family planning methods has demonstrated that increasing the number of contraceptive products yielded increases in uptake and protection from unintended pregnancy (31, 32). New PrEP products, particularly longer-acting PrEP, could reduce challenges with oral PrEP persistence and adherence and may be convenient for some women to use. Newer PrEP products may also have less effect on bone density, making them a good alternative for women worried about BMD loss during pregnancy. However, safety data on the use of these products by pregnant and breastfeeding women are still forthcoming and the current product labels exclude their use by these populations.

We acknowledge that our study has several limitations. First, we used pill count as a measure of adherence which might not accurately reflect whether participants adhere to PrEP or not. Adherence measured using pill counts does not always align with TFV levels measured using pharmacologic adherence measures such as plasma and dried blood spots (DBS) (33–36). However, pharmacologic methods require skilled laboratory personnel and specialized equipment, making them difficult to access in resource-limited settings such as Uganda (37). A point-of-care TFV (POC TFV) urine test could be used for data-driven adherence counseling to support young women using PrEP (38–40). Future studies are planned to evaluate PrEP exposure using POC TFV (41). Even with these limitations, PrEP adherence was relatively poor during pregnancy in our study population, and future studies should evaluate the impact of more consistent TDF-based PrEP exposure on BMD decline during pregnancy.

Second, we used DXA scans at enrollment and exit from the study. For some women, the exit DXA scan closely followed the end of pregnancy while for others the length of time between pregnancy and the DXA scan was longer. BMD begins to rebound after pregnancy and continues to rebound after breastfeeding ceases and thus, the longer the interval between the end of pregnancy and the exit DXA scan, the greater the potential for lactation to confound the relationship between PrEP, pregnancy, and BMD since most women in Uganda aim to breastfeed for 2 years. Our data on breastfeeding were insufficient to accurately account for the effect of lactation. Additionally, our analysis did not account for the length of PrEP exposure during pregnancy. The extent of bone loss could be different between those with longer-term PrEP exposure compared to women with shorter-term PrEP exposure.

## Conclusions

In conclusion, we found that BMD decline during pregnancy was not significantly greater among women who used PrEP during pregnancy compared to pregnant women with no PrEP exposure, suggesting that BMD loss in PrEP-using pregnant women is largely driven by pregnancy rather than PrEP use. Our study has also shown that women who experienced pregnancy while using PrEP were less likely to adhere to or continue using PrEP than those who did not experience pregnancy. Taken together, further assessments of the effect of quantifiable TDF-based PrEP use during pregnancy on bone health are needed. Additionally, it is important to advance research on alternative PrEP products that may have a lesser effect on bone health and could improve PrEP adherence during pregnancy.

## Data Availability

Public sharing of individual participant data was not included in the informed consent form of the project and cannot be posted in a supplemental file or a public repository because of legal and ethical restrictions. De-identified data underlying this project can be made available to interested researchers upon reasonable request by contacting the corresponding author.
